# Incorrect Disclosure and Role of Funder Statements

**DOI:** 10.1001/jamanetworkopen.2024.23203

**Published:** 2024-06-10

Errors were introduced during the editing process for the Original Investigation, “Efficacy and Safety of 5-Day Oral Ensitrelvir for Patients With Mild to Moderate COVID-19: The SCORPIO-SR Randomized Clinical Trial,”^1^ that was published on February 9, 2024, in *JAMA Network Open*, and that resulted in incorrect role of funder and disclosure statements. The disclosure statement for the first author, Hiroshi Yotsuyanagi, MD, PhD, should have been “Dr Yotsuyanagi reported receiving consulting fees, lecture fees, and travel support from Shionogi & Co, Ltd and lecture fees from MSD, Pfizer, and ViiV Healthcare.” The role of the funder statement should have been “Author employees of Shionogi & Co, Ltd participated in the design and conduct of the study; collection, management, analysis, and interpretation of the data; preparation, review, and approval of the manuscript; and decision to submit the manuscript for publication.” The article has been corrected.^1^
